# Molecular and Functional Insights into Thyrostimulin and Its Subunits-GPA2/GPB5

**DOI:** 10.3390/ijms262311523

**Published:** 2025-11-27

**Authors:** Nasreen Nakad, Fuad Fares

**Affiliations:** Department of Human Biology, University of Haifa, Mount Carmel, Haifa 3498838, Israel; nasreen.nakad@gmail.com

**Keywords:** glycoprotein hormones, thyrostimulin, GPA2 subunit, GPB5 subunit, TSH, TSH receptor

## Abstract

Thyrostimulin is a heterodimeric glycoprotein hormone composed of two subunits, GPA2 and GPB5, first identified in 2002. It is considered an ancestral member of the glycoprotein hormone family and is highly conserved across species, including vertebrates and invertebrates. Unlike classical pituitary glycoprotein hormones such as TSH, LH, and FSH, thyrostimulin appears to function predominantly through paracrine and autocrine mechanisms, with its expression reported in diverse tissues such as the pituitary, ovary, skin, and brain. In humans, thyrostimulin has been implicated in ovarian cancer cell proliferation, stem cell quiescence in the pituitary, and metabolic regulation. However, its role in metabolism remains unclear, with studies showing both beneficial and adverse effects such as weight loss in some models and elevated levels in polycystic ovary syndrome and metabolic syndrome patients. In *Caenorhabditis elegans*, orthologs of GPA2 and GPB5 have been shown to influence growth and intestinal function via a neuroendocrine pathway involving thyrotropin hormone-like peptides. These findings suggest that thyrostimulin has conserved multifunctional roles in development, metabolism, and endocrine signaling. The aim of this review is to summarize the structure–function relationships and the currently known roles of thyrostimulin and its subunits, GPA2/GPB5, particularly in the reproductive system, metabolic syndrome, skeletal development, and obesity.

## 1. Introduction

Thyrostimulin is a heterodimeric glycoprotein hormone composed of two subunits: α2 (GPA2) and β5 (GPB5), and it is highly conserved across species [1,2,3]. Its basic structure resembles that of human pituitary glycoprotein hormones (GPHs), follitropin (FSH), lutropin (LH), and thyrotropin (TSH) as well as placental human chorionic gonadotropin (hCG), all of which are heterodimeric hormones consisting of α (GPA1) and β (GPB1) subunits [4].

It has been reported that GPA2/GPB5 heterodimers may act locally, suggesting a paracrine rather than an endocrine role for thyrostimulin [2,5]. Co-expression of GPA2 and GPB5 has been detected in the pituitary [1,6,7], ovary [8,9], and skin [10]. However, some studies indicate that certain species do not produce heterodimeric thyrostimulin [11], and thus, the functional role of thyrostimulin and/or its subunits remains unclear.

Thyrostimulin binds to and activates the thyroid-stimulating hormone receptor (TSHR) with even greater binding affinity than TSH itself [6,8]. It shares a binding epitope with TSH on the extracellular domain of TSHR and activates the receptor via coupling with the G protein α subunit [6,12], leading to activation of adenylate cyclase and increased intracellular cAMP levels. Therefore, thyrostimulin may be involved in the hypothalamic–pituitary–thyroid (HPT) axis. However, its presence in various tissues across different species suggests that it may also have multifunctional roles.

Despite extensive studies, the effects and signaling pathways of thyrostimulin remain unclear and inconclusive. Research has investigated its presence and function in the pituitary gland, pituitary stem cells, female reproductive system pathologies, skeletal development, and obesity. Additionally, studies in species such as *Caenorhabditis elegans* (*C. elegans*) have explored its role in enteric neurons, glial cells, and the intestine.

The purpose of this review is to summarize the structure–function relationships and the currently known roles of thyrostimulin and its subunits, GPA2/GPB5, particularly in the reproductive system, metabolic syndrome, skeletal development, and obesity.

## 2. Thyrostimulin Structure

Thyrostimulin is a heterodimeric glycoprotein hormone composed of two subunits: GPA2 and GPB5. Its basic structure resembles that of human GPHs. Sequence analysis reveals that GPA2 shares only 35% sequence identity with GPA1, while GPB5 shares 37–42% identity with GPB1 [1,2]. As a heterodimer, thyrostimulin consists of approximately 240 amino acids (AAs) in total. GPA2 comprises 129 AAs, including a 23-AA signal peptide, resulting in a mature form of 106 AAs. GPB5 consists of 120–129 AAs, including a signal peptide [2].

Further structural differences have been identified when comparing thyrostimulin subunits with TSH hormone subunits. Like other GPH subunits, thyrostimulin and TSH belong to the superfamily of cystine-knot core motif proteins [3,5,13]. However, a key distinction is the absence of the cysteine residues necessary for forming the fastener in the seatbelt mechanism within the GPB5 subunit, although it retains all the cysteine residues required for cystine knot formation.

The seatbelt mechanism is vital for the stability of glycoprotein hormone heterodimers, as it involves a segment of the β subunit wrapping around the α subunit and being secured by a disulfide bridge between two cysteine residues on the β subunit. The absence of the conserved ‘seatbelt’ cysteines in GPA2/GPB5 likely reduces the thermodynamic stability of the heterodimer compared to classical glycoprotein hormones, suggesting that any functional complex may form transiently or locally. Therefore, current evidence supports the possibility of GPA2/GPB5 heterodimer formation but leaves its stability and in vivo persistence unresolved. This uncertainty is consistent with a model in which thyrostimulin acts in a paracrine or autocrine manner rather than as a stable, circulating endocrine hormone [1,3,5,14,15,16].

Despite this structural limitation, studies on a mutated hCG*β* subunit of similar length to GPB5, which lacks the sequence encoding the seatbelt region after cys100 in the C-terminal, demonstrate its ability to heterodimerize. These findings support the hypothesis that thyrostimulin subunits can form heterodimers even in the absence of the seatbelt mechanism [17]. Furthermore, it was proposed that thyrostimulin subunits are likely to heterodimerize when present at suitable concentrations. This observation suggests a short-term role for thyrostimulin, as hormonal concentrations are typically diluted in systemic circulation [5].

Both thyrostimulin subunits, GPA2 and GPB5, contain N-linked oligosaccharide chains, which have an important role in hormone expression as well suggesting a crucial role in its ability to activate the receptor. In GPA2 subunit, two N-linked oligosaccharide chains express at Asn14 and Asn58 sites, different from GPA1, which express the chains at Asn52 and Asn78 [4,14,18]. It was reported that glycosylation is critical for correct expression and secretion: mutants lacking both GPA2 glycosylation sites, or the GPB5 glycosylation site, fail to be secreted or even expressed in cells [3,14]. Disruption in one of the GPA2 oligosaccharide specifically Asn58 decreases receptor activation, and the simultaneous disruption of both chains results in a complete loss of GPA2 expression in cell lysates and media [4,14,18]. On the other hand, GPB5 contains a single N-linked oligosaccharide chain at Asn63 near the N-terminus, at the same position of TSHβ but distinct from other GPHs. Deleting the Asn63 chain on GPB5 resulted in the inhibition of the β subunit expression both in cell lysate and media [14].

Similarly, it was reported that N-linked oligosaccharides play an important role in TSH, FSH and hCG function. Deletion of N-linked oligosaccharides reduced assembly of α and β subunits; however, it does not affect binding to the receptor, but it significantly reduces bioactivity [18,19,20,21,22,23].

These findings emphasize the role of oligosaccharides in the expression and function of GPHs as well as thyrostimulin. This is consistent with earlier research showing that deletion of the oligosaccharide chain in the TSHβ subunit caused a drastic reduction in its expression, with more than 90% of TSH synthesis being diminished [24].

## 3. Thyrostimulin Binding Site

Thyrostimulin was initially thought to only activate TSHR, but not other glycoprotein hormone receptors such as FSH (FSHR) or LH (LHR) receptors, as demonstrated in nearly all published studies. However, it was recently discovered that single-chain thyrostimulin in the small-spotted catshark (*Scyliorhinus canicula*) (Sc) exhibits broader receptor specificity, activating not only ScTSHR but also ScFSHR, and ScLHR [25]. These findings derive from a limited set of species-specific experiments and recombinant constructs.

Interestingly, thyrostimulin activates TSHR with higher affinity than TSH in certain tissues. Thyrostimulin dimer is suggested to bind two distinct sites on TSHR as was observed by experimental observation [6]. This Hypothesis is supported by results demonstrating TSH hormone inability to compete with thyrostimulin for TSHR binding, indicating distinct binding sites on the receptor. However, the potent competition observed between thyrostimulin and TSH for TSHR binding site of TSH hormone implies a partial overlap between the binding sites of the two hormones. Collectively, these observations support the presence of a common binding site on TSHR, as well as a second site specific to thyrostimulin [6].

Furthermore, the inability of TSH to compete with thyrostimulin for TSHR binding could be attributed to posttranslational modifications of TSHR, which might result in the generation of receptor variants possessing distinct ligand-binding sites [6,26]. These modifications could impact the oligomeric state or conformational dynamics of TSHR, influencing its affinity and interaction with various ligands, including TSH, TSHβ variant, and thyrostimulin [6,26,27,28,29].

## 4. Thyrostimulin Signal Transduction

Thyrostimulin binds to TSHR and activates the target cells through G protein-coupled receptors (GPCRs). Like other glycoprotein hormone receptors (GPHRs), TSHR belongs to the leucine-rich repeats containing receptors, characterized by a long extracellular domain that facilitates the binding of a large heterodimer ligands [30].

Upon activation, TSHR stimulates several intracellular signaling cascades by activating different downstream G-proteins pathways. The classic signaling pathway, generally activated by TSH in pituitary cells, mediates the activation of Gαs-cAMP/protein kinase A/ERK pathway. However, under specific conditions such as activation by TSHR antibodies in Graves’ disease, TSHR signaling is mediated through Gαq/11. This leads to phospholipase C (PLC) activation, which subsequently induces the release of intracellular calcium, nuclear factor of activated T-cells (NFAT) activation, and the stimulation protein kinase C (PKC) and MAP kinase (MAPK) signaling pathways [1,31,32].

Interestingly, TSHR expression has been detected in various unexpected peripheral tissues beyond the thyroid gland, including bone marrow, adipose tissue, brain, and ovaries. In these tissues, TSHR can be activated by locally produced and secreted ligands such as thyrostimulin and TSHβ-variant, which has been shown to influence tissue function and development. This widespread expression suggests that TSHR plays broader physiological roles beyond its primary involvement in thyroid regulation. For example, thyrostimulin and its subunits are believed to have crucial roles in temporary developmental processes. These include the regulation of progenitor and stem cell differentiation, self-renewal, and survival [6,8,9,33,34,35].

Supporting this, recombinant TSH has been shown to enhance the differentiation of mesenchymal stem cells from human bone marrow into the chondrogenic cell lineage as well, suggesting the presence of local TSHR effectors regulating tissue development and repair [36].

## 5. Evolutionary Conservation of Thyrostimulin

Thyrostimulin is believed to represent the ancestral glycoprotein hormone, as its subunits GPA2 and GPB5 are highly conserved across both vertebrate and invertebrate species. According to phylogenetic studies, GPA2 and GPB5 subunits orthologs originated for more than 1 billion years ago, even before the divergence of nematodes, arthropods and vertebrates [2,33,37].

In invertebrates, it has been detected in arthropods, including *Drosophila*, *Rhodnius prolixus*, and mosquitoes [37,38,39,40]; nematodes such as *C. elegans*; bilaterian metazoans [2,41,42]; annelids such as the marine *Platynereis* [43]; mollusks, including the lophotrochozoan *Aplysia*, marine cone snails, and *Lottia gigantea* [44,45,46]; urochordates such as *Ciona intestinalis* [47]; and cephalochordates such as amphioxus [48,49].

In amphioxus, a member of the cephalochordates a subphylum within the phylum Chordata, thyrostimulin is the only glycoprotein hormone expressed. Notably, the phylum Chordata represents an evolutionary bridge between invertebrates and vertebrates, emphasizing thyrostimulin’s critical physiological role, especially in invertebrates, as well as its identity as the ancestral glycoprotein hormone [48,50].

This is further supported by the high homology of *GPA2* and *GPB5* orthologs expressed in *Drosophila* with mammalian subunits, where their heterodimerization has been shown to activate a fly G protein-coupled receptor containing leucine-rich repeats, which is highly homologous to the mammalian TSHR [8,37]. In contrast, other glycoprotein subunits, such as *GPA1* and the hormone-specific *GPB* subunits, exhibit conservation that is highly restricted to vertebrates [41,51]. This broad evolutionary presence of *GPA2* and *GPB5* highlights thyrostimulin’s ancient role in glycoprotein hormone signaling.

## 6. Comparative Analysis of the Functional Roles of Thyrostimulin and TSH

Both Thyrostimulin and TSH have been identified as ligands for TSHR. However, multiple studies showed that these two hormones are produced and secreted under distinct regulatory mechanisms, each fulfilling unique biological roles due to differences in their localization. Thyrostimulin is expressed in corticotrophs cells within the anterior pituitary and in diverse peripheral tissues such as skeleton, testis, skin, retina and much more while the TSH is expressed in the pituitary thyrotroph cells only. Further distinct difference is the hormones’ ability to bind and activate the human TSHR as each of the thyrostimulin subunits GPB5 and GPA2 alone were shown to be an independent ligand, without forming a heterodimer hormone complex [1,2,6,15,52]. This contrasts with TSH, which requires dimerization for its secretion and activation [4,21,53]. The ability of both TSH and thyrostimulin to activate only TSHR in humans suggests that thyrostimulin may function as an alternative ligand, as its receptor activation occurs independently of the classical thyrotropin-releasing hormone (TRH)-mediated TSH secretion pathway [6,52]. Unlike the TSH release mechanism, thyrostimulin’s activation of TSHR is independent of TRH signaling. More recently, in *Scyliorhinus canicula*, thyrostimulin subunits and a single-chain construct of ScGPB5–ScGPA2 were shown to activate not only ScTSHR but also ScFSHR and ScLHR, indicating broader receptor promiscuity in early vertebrates [25]. This demonstration that recombinant single-chain ScGPB5–ScGPA2 can activate multiple elasmobranch glycoprotein hormone receptors (ScTSHR, ScFSHR, and ScLHR) provides valuable insight into the functional plasticity of ancestral receptor–ligand systems [25]. However, this pleiotropic activation should be interpreted cautiously in the context of receptor evolution. Elasmobranch glycoprotein hormone receptors represent an early vertebrate lineage, preceding the sequence divergence and domain specialization that conferred tighter ligand specificity in *tetrapods*. Comparative analyses indicate that critical residues in the leucine-rich repeat domain and hinge region, which determine ligand selectivity in mammalian receptors, are not fully conserved in chondrichthyans. Thus, receptor promiscuity observed in sharks likely reflects ancestral receptor architecture rather than a direct functional parallel to mammalian systems. Understanding these evolutionary distinctions is essential for accurately interpreting cross-species findings and for assessing whether thyrostimulin–TSHR interactions in mammals retain vestiges of this ancestral versatility.

The effects of the two hormones also differ, as thyrostimulin activity appears to vary significantly across different models. In some studies, deletion of the hormone or its subunits did not affect mature organisms. For instance, deletion of *GPHB5* in mice showed no significant phenotypic changes, whereas in other organisms, such as *C. elegans*, its deletion affected normal body size development [6,8,41]. In contrast, patients with mutations in the TSHβ subunit exhibited defective TSH bioactivity, which was associated with congenital traits [54].

## 7. Function of Thyrostimulin and Its Subunits

Over the years, numerous studies on thyrostimulin have revealed its widespread expression in various tissues and its broad spectrum of functions, including stimulation of ovarian cancer progression, increased metabolic rate leading to weight loss, thyroid gland stimulation, and regulation of bone formation during skeletal development [1,8,9,33,35]. Together, these findings highlight the hormone’s significant role in both normal physiological processes and disease mechanisms. A summary of thyrostimulin’s effects across cell lines and experimental models is presented in Table 1.

### 7.1. Functional Role of GPHA2 in Pituitary Gland Development

GPHA2 is expressed in the anterior pituitary, particularly in non-endocrine cells, including pituitary stem or progenitor cells [34]. Its expression in these stem-like cells suggests it plays a key role in maintaining glandular homeostasis during both development and adult life. GPHA2 appears to support pituitary development by acting as a local signal involved in stem cell maintenance, tissue organization, and possibly in the regulation of endocrine progenitor differentiation.

Single-cell transcriptomic analyses have identified GPHA2 as a marker enriched in SOX2 positive pituitary stem cells, a population known to be regulated by NOTCH signaling [34,55]. Co-expression patterns and functional clustering analyses further suggest that GPHA2 expression may be regulated by NOTCH2 activity, linking this glycoprotein hormone subunit to pathways involved in maintaining stem cell quiescence and mediating local signaling within the anterior pituitary.

GPHA2 has been hypothesized as a downstream target of the NOTCH2 signaling pathway, particularly in the context of pituitary stem cell maintenance [34]. NOTCH2 activity is known to play a crucial role in the maintenance, differentiation, and proliferation of pituitary stem cells. These findings support a potential role for GPHA2 as a mediator of NOTCH2 driven signaling networks during pituitary development and homeostasis.

Treatment of dissociated adult pituitary cells was reported to increase the activity of cAMP Response Element-Binding Protein (CREB), a downstream target of TSHR an effect that was reversed by a TSHR inhibitor. These findings suggest that GPHA2 may function as a stem cell factor connected to the NOTCH2 signaling pathway and may be capable of activating TSHR signaling [34].

Although pituitary stem cells themselves do not express TSHR, GPHA2 is secreted within the pituitary and could act as a paracrine factor on neighbouring progenitor cells. This implies a potential role in regulating pituitary development by influencing progenitor differentiation. Alternatively, GPHA2 may play a more direct role in maintaining the quiescence of pituitary stem cells. This idea is supported by studies in the cornea, where GPHA2 was identified as a marker for quiescent corneal stem cells [34,56], and by further evidence suggesting its role in preserving the undifferentiated state of corneal stem cells [57]. Taken together, these findings suggest a dual role for GPHA2 in the pituitary: (1) maintaining stem cell quiescence and (2) acting as a paracrine signal influencing neighboring progenitor cells. Further investigation is needed to elucidate the direct roles of GPHA2 and NOTCH2 in sustaining the undifferentiated, quiescent state of pituitary stem cells [34].

### 7.2. The Emerging Role of Thyrostimulin in the Reproductive System

Over the years, glycoprotein hormones such as FSH and LH, along with their receptors, have been extensively studied for their roles in the reproductive system. However, the expression and function of TSHR and its potential ligands, such as thyrostimulin, within the reproductive system have only been explored more recently. Expression of TSHR and thyrostimulin in mammalian ovaries was detected only in the past decade and a half. Thyrostimulin expression has primarily been found in oocytes, suggesting it may play a primitive role in reproductive physiology [8,58].

Additionally, expression of an endogenous and functional TSHR has been reported in human epithelial ovarian cancer (EOC) cells [8].

Studies investigating thyrostimulin’s interaction with TSHR in human OVCAR-3 cells demonstrated its ability to promote cell proliferation by activating the cAMP cascade. This activation triggered cross-communication with the ERK and AKT signaling pathways, primarily through EGFR transactivation, and was shown to be PKA-independent.

These results were further supported by the use of adenylyl cyclase, MEK, or PI3K inhibitors, all of which blocked thyrostimulin-induced cell proliferation [9,59]. EGFR inhibition also reduced thyrostimulin’s ability to promote EOC cell proliferation, halting AKT phosphorylation and partially reducing ERK phosphorylation. However, EGFR inhibition did not fully suppress thyrostimulin-induced ERK activation, suggesting the involvement of alternative downstream effectors of TSHR in ERK signaling.

These findings align with prior research highlighting the role of cAMP in EGFR transactivation across various cancer types [60], as well as the involvement of the MEK–ERK pathway in regulating cell proliferation in multiple cancers [61]. The MEK–ERK pathway is known to be directly stimulated by the cAMP cascade [59], which is the primary signaling pathway activated by gonadotropins. Gonadotropins have been shown to induce ERK activation via calcium- and PKCδ-dependent mechanisms, thereby promoting the proliferation and migration of epithelial ovarian cancer cells. Thus, it is possible that thyrostimulin-TSHR signaling may function similarly to other gonadotropins in promoting ovarian cancer progression [9,62,63].

Thyrostimulin-like molecules were found in many vertebrates and invertebrates species like Drosophila melanogaste, amphioxus, and *C. elegance* suggesting that thyrostimulin predates the emergence of gonadotropin genes, representing the ancestral origins of glycoprotein hormones [37,41,48]. This emphasizes that the early functions of the thyrostimulin molecule orthologs included roles in reproductive control, gonadal development, metabolic rate and other developmental aspects. Interestingly, in fish ovaries, TSHR expression is temporally regulated. In catfish, TSHR levels peak after the vitellogenic growth phase, with a significant increase during the spawning period, followed by a gradual decline until the subsequent phase of ovarian recrudescence [64]. This regulation is consistent with other findings regarding its ligand, thyrostimulin, which has transient and significant function during early stages of skeletal development [33]. Furthermore, the presence of functional TSHR in mature human granulosa cells suggests that it plays a regulatory role in ovarian activity [8]. This indicates that TSHR not only contributes to the development and maintenance of ovarian function but also may influence the fine-tuning of ovarian responses to hormonal signals during different stages of ovarian cycle. In mammalian ovaries TSHR levels and activity during ovarian development were found to be thoroughly regulated by gonadotropin signaling. Gonadotropins receptor activation leading to an increased level of the secondary messenger cAMP, which in turn upregulates TSHR transcription, supported by the presence of a functional cAMP response element located between 131–139 bp upstream the human TSHR gene’s translational initiation site, a region conserved across mammals. Conversely, TSHR levels are downregulated by elevated estradiol levels, which increase in response to gonadotropins stimulation during follicle growth and luteinization. Furthermore, isolated rat granulosa cells from different ovarian stages treated with recombinant thyrostimulin heterodimer expressed significant increase in cAMP levels and activation of c-fos gene oncogene, in the presence of gonadotropins. These findings point to a paracrine system where thyrostimulin, expressed and secreted by oocytes, stimulates granulosa cells that express functional TSHR [8]. This highlights the transient nature of TSHR expression and the crucial role of gonadotropins in modulating ovarian TSHR levels throughout the ovarian development [8].

### 7.3. Association of GPHB5 with Insulin Resistance in Women with Polycystic Ovary Syndrome

Polycystic ovary syndrome (PCOS) affects approximately 5–12% of women of reproductive age worldwide. Its diagnosis is typically based on clinical features such as oligovulation or anovulation, the presence of polycystic ovaries, and hyperandrogenism, among other criteria. Studies over the years shown that nearly 70% of women with PCOS experience insulin resistance (IR) and associated metabolic disturbances. Although IR and metabolic dysfunction are highly prevalent in PCOS, they are not yet formally included in the diagnostic guidelines [65,66,67,68]. Beyond reproductive complications, PCOS is associated with an increased risk of obesity and a range of metabolic conditions, including type 2 diabetes, atherosclerosis, metabolic syndrome, and cardiovascular diseases [65,69,70,71,72].

Through bioinformatic analysis, it wasdentified that *GPHB5* was a gene associated with PCOS and metabolic disorders [65]. The study emphasized that the expression of genes related to glucose and lipid metabolism was elevated within the activated GPHB5 signaling pathway. These genes include adrenoceptor alpha 2A (ADRA2A), growth differentiation factor 15 (GDF15), and BMP and activin membrane-bound inhibitor (BAMBI).

To further investigate the link between GPHB5 and metabolic regulation, GPHB5 mRNA levels were examined in both normal and obese mice. The results showed significantly elevated expression in key metabolic tissues such as skeletal muscle, adipose tissue, and liver in diet-induced obese and diabetic mice, as well as in rat models of PCOS, compared to wild-type (WT) controls [65].

In women with PCOS (195 patients) and IR (117 patients), circulating *GPHB5* levels were significantly elevated compared to healthy controls (171 controls). *GPHB5* levels showed a positive correlation with body mass index (BMI) and Homeostatic Model Assessment of Insulin Resistance (HOMA-IR), and a negative correlation with markers of insulin sensitivity, including the Euglycemic Hyperinsulinemic Clamp (EHC), M-values, and adiponectin levels. These findings reinforce the role of *GPHB5* in metabolic regulation and its association with insulin resistance [65]. However, these results contrast with an earlier study suggesting that *GPHB5* functions as a positive inducer of metabolism in diet-induced obesity models in mice [35].

### 7.4. GPHB5 and Its Relationship with Metabolic Syndrome in Young Women

In young women diagnosed with metabolic syndrome (MetS), circulating *GPHB5* levels have been proposed as a potential biomarker, as they were found to be significantly elevated compared to healthy controls [73]. Similarly, overweight and obese individuals exhibited higher circulating *GPHB5* levels and lower adiponectin concentrations relative to normal weight individuals, further supporting the association between *GPHB5* and metabolic dysregulation [73].

In the same study, *GPHB5* levels were positively correlated with several key MetS related parameters, including BMI, blood pressure, fasting blood glucose, fasting insulin, and free fatty acids (FFA). In contrast, negative correlations were observed between *GPHB5* levels and both high-density lipoprotein cholesterol (HDL-C) and adiponectin levels.

The consistent elevation of *GPHB5*, alongside increased markers of MetS, suggests its involvement in long term metabolic regulation rather than in acute metabolic responses. Supporting this, *GPHB5* levels remained unchanged during oral glucose tolerance tests where its expression is not acutely regulated by short term fluctuations in blood glucose, insulin, or FFA levels [73]. Furthermore, treatment of MetS patients with glucagon-like peptide-1 receptor agonists (GLP-1RA) over a six-month period led to a significant reduction in serum *GPHB5* levels after 3 to 6 months of therapy. This reduction was accompanied by marked weight loss, improved glucose and lipid metabolism, and enhanced insulin sensitivity, as indicated by increased levels of adiponectin an insulin sensitizing hormone [73].

Collectively, these findings suggest that *GPHB5* is associated with impaired glucose and lipid metabolism and insulin resistance, potentially functioning as a negative regulator of metabolic homeostasis. Its expression appears to be influenced by the liver’s metabolic state in MetS patients, reinforcing the hypothesis that *GPHB5* may play a critical role in the pathophysiology of metabolic syndrome [73].

Although associations across large human cohorts are highly consistent, they remain correlational. Thus, the idea that GPHB5 acts as a negative regulator of metabolic homeostasis should be considered a working hypothesis, not a proven causal mechanism. These findings justify further mechanistic studies but do not, on their own, demonstrate direct regulatory action.

### 7.5. The Role of Thyrostimulin in Skeletal Development and Bone Physiology

Bone-related studies have indicated that thyrostimulin plays a significant role in skeletal development. This is supported by the expression of its subunits, *GPA2* and *GPB5*, in bone cells of both mice and humans. The *GPA2* subunit is expressed in osteoblasts and osteoclasts in both species. In contrast, *GPB5* is expressed at low levels only in mouse osteoblasts during early stages of differentiation, whereas in humans, *GPB5* mRNA is present in both osteoblasts and osteoclasts. Furthermore, mRNA for TSHR the targeted receptor of thyrostimulin is barely detectable in human osteoclasts but it is expressed in human and mouse osteoblasts, as well as in mouse osteoclasts [33].

In the skeletons of newborn mice, thyrostimulin expression was detected particularly in the skull and long bones, and it declined sharply in older mice with no activity observed in the adult skeleton [33]. These findings suggest that thyrostimulin serves a transient yet important role during early skeletal development. Further investigation using juvenile mice lacking thyrostimulin revealed enhanced bone volume and mineralization, attributed to increased osteoblastic bone formation. However, no cAMP response or activation of alternative signaling pathways such as Akt, ERK, or p38 MAP kinase was observed in primary calvarial osteoblasts or osteoblasts derived from bone marrow stromal cells. Additionally, in vitro studies showed that thyrostimulin failed to directly suppress osteoblast proliferation, differentiation, or mineralization. These findings suggest that thyrostimulin acts as an indirect negative regulator of osteoblastic bone formation during skeletal development [33].

In another study, it was reported that overexpression of *GPB5* in mice resulted in a shortened snout compared to wild-type controls. Specifically, the frontal and nasal bones both derived from neural crest ectoderm were reduced in length, while other bones of mesodermal origin remained unaffected [35,74].

The predicted structure of thyrostimulin in the skeleton, composed of both *GPA2* and *GPB5* subunits, appears to be less stable than other glycoprotein hormones [5]. This reduced stability supports the idea that thyrostimulin may function primarily as a local signaling molecule rather than a systemic hormone particularly given the limited understanding of its source cells and target sites in skeletal tissues [33].

These findings highlight thyrostimulin’s differential effects based on the embryonic origin of skeletal tissue, suggesting a complex and tissue specific role in skeletal development [35,74].

Consequently, further research is required to elucidate the specific mechanisms by which thyrostimulin influences skeletal development and to determine whether it has any residual role in adult bone physiology. A deeper understanding of these mechanisms could provide valuable insights into the evolutionary significance of thyrostimulin.

### 7.6. Thyrostimulin Orthologs Effect on Normal Development

In *C. elegans*, complete gene knockouts of both subunits, GPLA-2 and GPLB-5, showed a significant decrease in body length starting at day 1 of adulthood and persisting through day 5, implying that the reduction arises from impaired growth rather than a delay in developmental timing. Additionally, shorter body lengths were observed in different mutants of highly conserved region critical for cystine knot formation, or/and the lacking a large gene segment, including the start codon and first exon [41]. In this study, Kenis et al. also highlighted the targeted receptor importance as a loss of function mutant in FSHR-1 exhibited impaired growth that persisted into adulthood. And when expressing WT FSHR-1 the defect was completely rescued, providing compelling evidence that the receptor, along with its ligands GPLA-1 and GPLB-1, is indispensable for normal growth in *C. elegans* [41].

### 7.7. The Role of Thyrostimulin in Obesity

One of the pioneering studies investigating the origin and function of thyrostimulin β subunit (*GPB5*) was conducted [35]. This study aimed to elucidate the role of *GPB5* subunit, which the authors referred to as an “orphan glycoprotein hormone” (OGH). In this study, adult male and female homozygous mice lacking the *GPB5* subunit exhibited no significant phenotypic abnormalities, and heterozygous mice showed only minimal deletion of endogenous *GPB5* expression in certain tissues. However, in newborn mice, strong *GPB5* expression was observed in the salivary glands and in the palatal ducts of the palate. Despite this localized expression, there were no observable differences in body weight, body composition, or metabolic parameters compared to WT controls [35].

These findings suggest that *GPB5* may exert tissue specific functions, particularly in the salivary gland, an exocrine organ that may also exhibit mixed exocrine and endocrine activity. This supports the notion that thyrostimulin has multifunctional roles depending on tissue type. In contrast, mice with global transgenic overexpression of the *GPB5* subunit exhibited reduced body weight beginning at one month of age, which persisted into adulthood [35]. This further reinforces the idea that thyrostimulin may play distinct roles depending on both tissue context and developmental stage.

Further investigation into the observed weight reduction in mice overexpressing *GPB5* revealed significant differences in adiposity. Male transgenic mice subjected to a high fat diet exhibited only a 10–20% increase in body fat, compared to over 45% in WT controls. Interestingly, despite slightly higher food intake, these mice failed to gain weight, likely due to an elevated metabolic rate, as evidenced by increased oxygen consumption and carbon dioxide production [35].

The weight reduction in male mice was also associated with decreased blood glucose, triglyceride, and insulin levels, indicating enhanced insulin sensitivity and improved glucose metabolism. Moreover, both male and female mice overexpressing *GPB5* showed significantly lower body weight and serum cholesterol levels compared to WT controls. These metabolic changes suggest potential activation of extrathyroidal TSHR, which are known to be expressed in adipose tissue [1,35].

Hormonal analysis in these mice revealed a 1.5 to 2.5 fold increase in basal levels of circulating T3 and T4, with no change or even a reduction in TSH levels, likely due to negative feedback regulation [35]. These findings are consistent with earlier studies demonstrating that T3, via its nuclear receptor, directly regulates cholesterol metabolism [26,75].

Collectively, these studies demonstrate that thyrostimulin and its subunits play evolutionarily conserved and context-dependent roles in growth and metabolism. In *C. elegans*, disruption of thyrostimulin results in impaired somatic growth, emphasizing their essential contribution to normal developmental processes. In mammals, the subunit GPB5 appears to exert tissue-specific and metabolic effects, with overexpression leading to reduced body weight, enhanced metabolic rate, and improved insulin sensitivity, likely through activation of extrathyroidal TSHR signaling. Together, these findings highlight thyrostimulin as a multifunctional regulator that integrates developmental and metabolic pathways across species, and suggest that its activity is finely tuned according to tissue type and developmental stage.

## 8. Conclusions

Thyrostimulin, a heterodimeric glycoprotein hormone composed of GPA2 and GPB5, represents a distinct and evolutionarily conserved member of the glycoprotein hormone family that includes TSH, FSH, LH and hCG. Unlike classical pituitary hormones such as TSH which primarily function within the hypothalamic–pituitary–thyroid axis, thyrostimulin operates predominantly through autocrine and paracrine mechanisms across multiple tissues. By activating the TSH receptor and engaging the cAMP–PKA–CREB signaling cascade, it exerts regulatory control over gene expression, cell proliferation, and differentiation.

Accumulating evidence implicates thyrostimulin in a wide range of physiological processes, including reproductive regulation, metabolic homeostasis, and skeletal development, while findings from *C. elegans* highlight its conserved neuroendocrine functions across evolution. The requirement of *N*-linked glycosylation for its maturation and receptor activity further emphasizes the intricate post-translational control of this hormone’s function.

The divergent phenotypes in GPHB5 knockout versus overexpression models likely arise from a combination of factors. Compensatory pathways may develop in germline knockouts, differences in developmental timing alter how each model perturbs endocrine circuits, and strain-specific genetic backgrounds influence metabolic sensitivity. Together, these variables help explain the contrasting outcomes and highlight the complexity of comparing distinct model systems.

In summary, we propose three prioritized, testable hypotheses to guide the field of thyrostimulin research. First, GPB5 may act in an endocrine or paracrine manner depending on tissue context, a distinction that can be resolved using tissue-specific perturbations and quantitative measurements of local versus systemic GPB5. Second, monomeric GPB5 may retain intrinsic in vivo activity, which can be evaluated using dimerization-deficient mutants and structurally validated monomeric preparations. Third, GPB5 may engage TSHR through a binding interface that partially overlaps with the canonical TSH site, a hypothesis amenable to targeted mutagenesis and ligand-competition assays. Together, these focused questions provide a clear and tractable experimental roadmap for advancing the field.

## Figures and Tables

**Table 1:** Summary of Thyrostimulin Effects across Cell Lines and Experimental Models.

Species	Observed Effect	Reference
Human, Mice, Frogs, *C. elegans* & *Drosophila*	Identification of two novel human Glycoprotein Hormone Subunit Family genes, GPA2 and GPB5. Transcripts found in diverse human tissues (pituitary, brain). Immunoreactive GPA2 and GPB5 detected in the anterior pituitary of mice and frogs. Evolutionary conservation observed across multiple species (e.g., *C. elegans*, *Drosophila*), suggesting origin prior to bilaterian metazoans.	[2]
Human & Rats	GPA2 (α2) and GPB5 (β5) subunits identified as heterodimerization partners activating TSH receptors in vitro and in vivo. Both subunits colocalize in the rat anterior pituitary, indicating a potential paracrine signaling mechanism.	[1]
Mice	Male mice overexpressing GBP5: Weight reduction Decreased levels of blood glucose, triglycerides and insulin. Enhancement in insulin sensitivity and glucose metabolism. Female and Male mice overexpressing GBP5: Lower levels of serum cholesterol	[35]
Human, Mice, Chinese hamster & Baby hamster	Thyrostimulin expressed in corticotroph cells of the human anterior pituitary, skin, retina, and testis. More potent ligand of TSHR than TSH, with two binding sites (one shared with TSH, one unique). Mice overexpressing GPHB5 show reduced body weight, proptosis, and elevated T4.	[6]
Human & Rats	Discovery of an ovarian paracrine system where oocyte-derived thyrostimulin acts via TSHR in granulosa cells, tightly regulated by gonadotropins.	[8]
Human & Rats	Thyrostimulin promotes epithelial ovarian cancer (EOC) proliferation via trans-activation of the EGFR signaling pathway.	[9]
Human & Mice	GPAH2 controls the quiescent state of LSCs and is regulated by T cells.	[55]
Human & Mice	GPHB5 is a potential biomarker for metabolic syndrome (MetS). Circulating levels are elevated and correlate with obesity, hyperglycemia, hyperlipidemia, and hypertension. Acts as a negative regulator of glucose/lipid metabolism and insulin resistance.	[56]
Human, Rats and Mice	Circulating GPHB5 may serve as a biomarker and therapeutic target for PCOS and insulin resistance (IR). Elevated GPHB5 mRNA in metabolic tissues of obese/diabetic mice and PCOS rats; metabolic disorders and androgens stimulate its secretion.	[57]
*C. elegance* & Human	Discovery of an ancestral thyrostimulin-like neuroendocrine system regulating growth and intestinal function. GPA2/GPB5 orthologs signal via FSHR-1, essential for normal body size development.	[41]
Mice	GPAH2 identified as a NOTCH2-related stem cell factor that activates TSHR signaling, potentially impacting pituitary development.	[34]
*Elasmobranch* -*Scyliorhinus canicular* & Human	Thyrostimulin activates ScTSHR, ScFSHR, and ScLHR receptors, indicating broad receptor promiscuity in early vertebrates.	[25]

## Data Availability

No new data were created or analyzed in this study. Data sharing is not applicable to this article.

## Abbreviations

The following abbreviations are used in this manuscript:

GPHs	Human pituitary glycoprotein hormones
FSH	Follitropin
LH	lutropin
TSH	thyrotropin
hCG	Placental human chorionic gonadotropin
TSHR	Thyroid-stimulating hormone receptor
HPT	Hypothalamic pituitary thyroid
*C. elegans*	*Caenorhabditis elegans*
AAs	Amino acids
Sc	Scyliorhinus canicular
GPCRs	G protein-coupled receptor
GPHRs	Glycoprotein hormone receptors
PLC	Phospholipase C
NFAT	Nuclear factor of activated T-cells
PKC	Stimulation protein kinase C
MAPK	MAP kinase
TRH	Thyrotropin-releasing hormone
cAMP	cAMP Response Element-Binding Protein
EOC	Epithelial ovarian cancer
PCOS	Polycystic ovary syndrome
IR	Insulin resistance
GDF15	Growth differentiation factor 15
BAMBI	BMP and activin membrane-bound inhibitor
WT	Wild type
BMI	Body mass index
HOMA-IR	Homeostatic Model Assessment of Insulin Resistance
EHC	Euglycemic Hyperinsulinemic Clamp
MetS	Metabolic syndrome
FFA	Fatty acids
HDL-C	High-density lipoprotein cholesterol
GLP-1RA	Glucagon-like peptide-1 receptor agonists
OGH	Orphan glycoprotein hormone
HPT	Hypothalamic–pituitary–thyroid
